# The COVID-19 Pandemic Impact on Breast Cancer Diagnosis: A Retrospective Study

**DOI:** 10.1055/s-0042-1749207

**Published:** 2022-06-06

**Authors:** Erika Marina Solla Negrao, Cesar Cabello, Livia Conz, Edmundo Carvalho Mauad, Luiz Carlos Zeferino, Diama Bhadra Vale

**Affiliations:** 1 Cancer Prevention Institute, Hospital do Câncer de Barretos Barretos, SP, Brazil; 2 Obstetrics and Gynecology Department, Universidade Estadual de Campinas, Campinas, SP, Brazil

**Keywords:** breast neoplasms, early detection of cancer, quality of health care, COVID-19, mammography, neoplasias da mama, detecção precoce de câncer, qualidade da assistência à saúde, COVID-19, mamografia

## Abstract

**Objective** This study aimed to evaluate the diagnostic profile of breast cancer cases during the coronavirus disease 2019 (COVID-19) pandemic compared with the previous year.

**Methods** It is a retrospective study of cases diagnosed by a reference service in the public health system of Campinas, SP, Brazil. Two periods were analyzed: March to October 2019 (preCOVID period) and March to October 2020 (COVID-period). All women diagnosed during the periods were included. The Chi-Squared or Fisher exact and Mann-Whitney tests were used.

**Results** In the preCOVID and COVID periods, breast cancers were diagnosed, respectively, in 115 vs 59 women, and the mean ages at diagnosis were 55 and 57 years ( *p* = 0.339). In the COVID period, the family history of breast cancer was more observed (9.6% vs 29.8%, *p* < 0.001), cases were more frequently symptomatic (50.4% vs 79.7%, *p* < 0.001) and had more frequently palpable masses (56.5% vs 79.7%, *p* = 0.003). In symptomatic women, the mean number of days from symptom to mammography were 233.6 (458.3) in 2019 and 152.1 (151.5) in 2020 ( *p* = 0.871). Among invasive tumors, the proportion of breast cancers in stages I and II was slightly higher in the COVID period, although not significantly (76.7% vs 82.4%, *p* = 0.428). Also in the COVID period, the frequency of luminal A-like tumors was lower (29.2% vs 11.8%, *p* = 0.018), of triple-negative tumors was twice as high (10.1% vs 21.6%, *p* = 0.062), and of estrogen receptor-positive tumors was lower (82.2% vs 66.0%, *p* = 0.030).

**Conclusion** During the COVID-19 pandemic, breast cancer diagnoses were reduced. Cases detected were suggestive of a worse prognosis: symptomatic women with palpable masses and more aggressive subtypes. Indolent tumors were those more sensitive to the interruption in screening.

## Resumo

**Objetivo** Este estudo teve como objetivo avaliar o perfil diagnóstico dos casos de câncer de mama na pandemia de *coronavirus disease 2019* (COVID-19) em comparação com o ano anterior.

**Métodos** Este é um estudo retrospectivo de casos diagnosticados em um serviço de referência da rede pública de saúde de Campinas, SP, Brasil. Foram analisados dois períodos: de março a outubro de 2019 (período pré-COVID) e de março a outubro de 2020 (período COVID). Todas as mulheres diagnosticadas durante os períodos foram incluídas. Foram utilizados os testes do qui-quadrado ou exato de Fisher e Mann-Whitney.

**Resultados** Nos períodos pré-COVID e COVID, o câncer de mama foi diagnosticado, respectivamente, em 115 e 59 mulheres, e a média de idade no diagnóstico foi de 55 e 57 anos ( *p* = 0,339). No período COVID, foram mais frequentes a história familiar de câncer de mama (9,6% vs 29,8%, *p* < 0,001), casos sintomáticos (50,4% vs 79,7%, *p* < 0,001) e com massas palpáveis (56,5% vs 79,7%, *p* = 0,003). Nas mulheres sintomáticas, a média de dias desde os sintomas até a mamografia foi de 233,6 (458,3) no pré-COVID e 152,1 (151,5) no COVID ( *p* = 0,871). Entre os tumores invasivos no período COVID, a proporção de cânceres nos estágios I e II foi ligeiramente maior, porém não significativa (76,7% vs 82,4%, *p* = 0,428). Ainda no período COVID, a frequência de tumores tipo *luminal A-like* foi menor (29,2% vs 11,8%, *p* = 0,018), de tumores triplo-negativos foi duas vezes maior (10,1% vs 21,6%, *p* = 0,062), e de tumores positivos para receptor de estrogênio foi inferior (82,2% vs 66,0%, *p* = 0,030).

**Conclusão** Durante a pandemia de COVID-19, houve uma redução no diagnóstico de câncer de mama. Os casos detectados eram sugestivos de pior prognóstico: mulheres sintomáticas com massas palpáveis e subtipos mais agressivos. Os tumores indolentes foram os mais sensíveis à interrupção do rastreamento.

## Introduction

The coronavirus disease 2019 (COVID-19) pandemic has had unprecedented effects on healthcare systems worldwide, and the impact on cancer care is being predicted from the outset. 1 Studies in different countries have already demonstrated its effects, with delays in detecting and treating breast cancer. 2 3 4

At the beginning of the pandemic, there were international recommendations to discuss the option of postponing surgical treatment until the pandemic was under better control, according to the risk/benefit ratio. In Brazil, the recommendation published by the Society of Clinical Oncology in May 2020 recommended for newly diagnosed breast cancer, initiation of systemic treatment with neoadjuvant endocrine therapy or neoadjuvant chemotherapy, with anti-HER2 blockade if the disease was HER2 positive. In postmenopausal patients and luminal A-like cancer cases, including clinical stages I and II, neoadjuvant endocrine therapy and surgery after 3 to 4 months, or when appropriate, if responsive, were options to be considered. 5

In Brazil and other middle-income countries, there is inequity in access to screening tests, cancer detection and breast cancer treatment. 6 7 In March 2020, Brazilian medical societies launched a joint note to stop screening evaluations due to the COVID-19 pandemic announcement. Diagnostic mammograms in symptomatic women should consider the degree of clinical suspicion and individual risk-benefit, with attention to patients over 60 years of age. 8 After July 2020, the Ministry of Health released a technical note recommending the return of actions observing a transitory relief on national indicators. 9

There is a suspicion that the pandemic would impact deepening inequalities in cancer care. 4 In diagnosis, it will lead to an upstaging of tumor worsening prognosis, reducing women's quality of life, demanding more resources to the health care system to treat more complex situations. 3 10 11 12 13

This study aimed to evaluate the diagnostic profile of breast cancer cases during the COVID-19 pandemic in users of the public health system in a populous urban city in Brazil, compared with the same period of the previous year. Clinical and pathological characteristics were analyzed. Diagnostic profiling studies can help to predict delays and better allocation of limited resources, targeting better effects on cancer outcomes, especially in public health systems.

## Methods

It is a retrospective descriptive study of breast cancer cases diagnosed by the leading reference service for diagnosing breast cancer in the public health system in Campinas, state of São Paulo, Brazil. Two periods were analyzed: March 1st to October 31st, 2019, preCOVID period, and March 1st to October 31st, 2020, when the COVID-19 worldwide pandemic was in full force. All women who were diagnosed at the service during the analyzed periods were included.

About half of the city's women depend exclusively on the public health system, which performs free-of-charge screening, diagnosis and treatment, at different levels of complexity of the services. 14 15 The request for screening mammography and the reception of symptomatic patients is performed in primary care. The reference service performs ∼ 70% of the mammograms, and ∼ 80% of breast cancer diagnoses in the city's public system, from biopsy to clinical staging. Positive cases are then referred to hospitals for treatment. During the pandemic, no modification was observed in the flow of patients. The incidence of invasive breast cancer in 2010 to 2014 in Campinas was 70.03 per 100,000 women. 16

Mammograms were performed using digital devices with complements. Biopsies were performed by core needle guided by ultrasound or stereotaxy. A central laboratory performs the histopathological and immunohistochemical analyses with direct links. The classification of tumor subtypes was based on hormone receptors (positive when above 1%), K _i_ -67% (positive when over 20%) 17 and HER2. The initial staging was performed by clinical evaluation by a breast cancer surgeon, complemented with imaging exams when necessary. The staging follows the AJCC clinical staging principles. 18

The study data were collected and managed through the service's information system, which uses electronic data capture via REDCap, hosted by the institution that maintains the unit. The compiled data form contains information about the patient's age (in complete years), presence or absence of symptoms, menopausal status and family history of breast cancer. Results of mammograms, biopsies, immunohistochemistry, HER2 and clinical staging were also registered.

To describe the sample profile according to the variables under study, frequency tables of categorical variables were drawn up, with absolute frequency (n) and percentage (%) values. Descriptive statistics of numerical variables were presented by mean and standard deviation (SD) values. To compare categorical variables between periods were used the Chi-squared or Fisher exact tests. To compare the numerical variables between periods, the Mann-Whitney test (2 groups) was used due to the absence of normal distribution of the variables. The significance level adopted for the statistical tests was 5%, that is, *p* < 0.05.

The ethics committee of the Universidade Estadual de Campinas, under the number CAAE 89399018.2.0000.5404 approved the study “Population-based assessment of breast cancer screening, diagnosis and death of women in the city of Campinas according to age”, ongoing since 2018. The Committee waived the need for consent due to the retrospective nature of the study.

## Results

From March to October 2019, preCOVID period, 115 women were diagnosed with breast cancer, and from March to October 2020, COVID period, 59 women were diagnosed. There was no significant difference in the mean age at diagnosis in the periods: 55 years in 2019 (santdard deviation [SD] = 10) and 57 years in 2020 (SD = 12) ( *p* = 0.339). The menopausal status of diagnosed women was not different between the periods ( *p* = 0.260). However, a family history of breast cancer was more present in the COVID period (9.6% in 2019 and 29.8% in 2020, *p* < 0.001) ( Table 1 ). Tumour detection mode changed over the period. In the pandemic, diagnosed cases were more frequently symptomatic (50.4% in 2019 and 79.7% in 2020, *p* < 0.001), women had more palpable masses (56.5% in 2019 and 79.7% in 2020, *p* = 0.003) and were diagnosed through diagnostic mammograms (47.0% in 2019 and 81.4% in 2020, *p* < 0.001). For symptomatic women, the mean days from symptom onset to mammography was 233.6 days (SD 458.3) in 2019 and 152.1 days (151.5) in 2020 ( *p* = 0.871) ( Table 1 ). The stage profile was not different between periods. Although fewer cases of in situ tumors were diagnosed, this difference was not significant (21.7% in 2019 and 13.6% in 2020, *p* = 0.193). Among invasive tumors, the proportion of breast cancers in stages I and II was slightly higher in the COVID period but not significant (76.7% in 2019 and 82.4% in 2020, *p* = 0.428) ( Table 1 ).

**Table 1:** Clinical and epidemiological analysis of breast cancer cases in a referral center in Campinas, Brazil, in the pre-COVID and COVID periods

Variables	2019 (pre-COVID)	2020 (COVID)	*P* -value*
	Average (SD)	Average (SD)	
Age in years	55.0 (10.2)	57.0 (12.0)	0.339
Time in days symptom to MMG ^†^	233.6 (458.3)	152.1 (151.5)	0.871
	**n (%)**	**n (%)**	
Total	115 (66.09)	59 (33.91)	
Age ^‡^			
40–49 years	32 (31.4)	13 (25.5)	0.452
50–69 years	70 (68.6)	38 (74.5)	
Menopause			
Yes	43 (37.4)	17 (28.8)	0.260
No	72 (62.6)	42 (71.2)	
Family history of breast cancer ^§^			
Yes	11 (9.6)	17 (29.8)	< 0.001
No	104 (90.4)	40 (70.2)	
Symptomatic			
Yes	58 (50.4)	47 (79.7)	< 0.001
No	57 (49.6)	12 (20.3)	
Palpable mass			
Yes	65 (56.5)	47 (79.7)	0.003
No	50 (43.5)	12 (20.3)	
Purpose			
Screening	61 (53.0)	11 (18.6)	< 0.001
Diagnosis	54 (47.0)	48 (81.4)	
Tumour invasive			
No (in-situ tumor)	25 (21.74)	8 (13.56)	0.193
Yes (stage I/IV)	90 (78.26)	51 (86.44)	
Stage			
Early (stage I + II)	69 (76.67)	42 (82.35)	0.428
Late (stage III + IV)	21 (23.33)	9 (17.65)	

Abbreviations: MMG, mammography; SD, standard deviation.

*P-value – Categoric variables: Chi-squared or Fisher test; numeric variables - Mann-Whitney test; ^†^ only from symptomatic tumors; ^‡^ for age analysis women younger than 40 or older than 69 years old were excluded; ^§^ missing information.

The subanalysis of the 110 cases diagnosed in stages I and II revealed a similar pandemic influence on the presentation of these cases. Mean age, menopausal status and time from symptoms to mammography was not statistically significant. However, as observed in the previous table, more women diagnosed in the COVID period had a family history of breast cancer (14.5% in 2019 and 31.7% in 2020, *p* = 0.032). The cases diagnosed were more frequently symptomatic (53.6% in 2019 and 90.5% in 2020, *p* < 0.001), women had palpable masses more frequently (65.2% in 2019 and 90.5% in 2020, *p* = 0.003) and were diagnosed through diagnostic mammograms (46.4% in 2019 and 90.5% in 2020, *p* < 0.001) ( Table 2 ).

**Table 2:** Clinical and epidemiological analysis of breast cancer cases diagnosed in Stages I and II, as a function of the pre-COVID and COVID period

Variables	2019 (pre-COVID)	2020 (COVID)	*P* -value
	Stage I + II	StageI + II	
	Average (SD)	Average (SD)	
Age in years	53.7 (10.2)	57.5 (12.3)	0.134
Time in days symptom to MMG ^†^	204.3 (492.5)	139.6 (145.3)	0.638
	**n (%)**	**n (%)**	
Total	69 (76.7)	42 (82.3)	
Age ^‡^			
40–49 years	21 (33.9)	8 (21.6)	0.195
50–69 years	41 (66.1)	29 (78.4)	
Menopause			
Yes	42 (60.9)	30 (71.4)	0.258
No	27 (39.1)	12 (28.6)	
Family history of breast cancer ^§^			
Yes	10 (14.5)	13 (31.7)	0.032
No	59 (85.5)	28 (68.3)	
Symptomatic			
Yes	37 (53.6)	38 (90.5)	< 0.001
No	32 (46.4)	4 (9.5)	
Palpable mass			
Yes	45 (65.2)	38 (90.5)	0.003
No	24 (34.8)	4 (9.5)	
Purpose			
Screening	37 (53.6)	4 (9.5)	< 0.001
Diagnosis	32 (46.4)	38 (90.5)	

Abbreviations: MMG, mammography; SD, standard deviation.

* *P* -value – Categoric variables: Chi-square or Fisher test; numeric variables - Mann-Whitney test; ^**†**^ only from symptomatic tumors; ^‡^ for age analysis women younger than 40 or older than 69 years old were excluded; ^§^ missing information.

Regarding the morphological characteristics of invasive tumors, the frequency of luminal A-like tumors was lower in the COVID period (29.2% in 2019 and 11.8% in 2020, *p* = 0.018). The frequency of triple-negative tumors was twice as high in the pandemic than in the pre-pandemic period (10.1% in 2019 and 21.6% in 2020, *p* = 0.062). The frequency of estrogen receptor-positive tumors in the pandemic was lower (82.2% in 2019 and 66.0% in 2020, *p* = 0.030). The other morphological characteristics evaluated did not show significant variation in the period ( Table 3 ).

**Table 3:** Analysis of the morphological characteristics of invasive breast tumors, as a function of the pre-COVID and COVID period

	2019 (pre-COVID)	2020 (COVID)	*P* -value*
	n (%)	n (%)	
Molecular subtype †			
Luminal A-like	26 (29.2)	6 (11.8)	0.018
Luminal B-like	37 (41.6)	24 (47.0)	0.529
Luminal HER	10 (11.2)	4 (7.8)	0.520
Triple-negative	9 (10.1)	11 (21.6)	0.062
Pure HER2	7 (7.9)	6 (11.8)	0.548
Notthingham †			
Grade I	13 (14.9)	9 (18.8)	0.230
Grade II	53 (60.9)	22 (45.8)	
Grade III	21 (24.1)	17 (35.4)	
Progesterone receptor			
Positive	56 (62.2)	27 (52.9)	0.282
Negative	34 (37.8)	24 (47.1)	
Estrogen receptor †			
Positive	74 (82.2)	33 (66.0)	0.030
Negative	16 (17.8)	17 (34.0)	
HER2 ^a^			
Positive	17 (19.1)	9 (17.7)	0.753
Negative	66 (74.2)	40 (78.3)	
Not conclusive	6 (6.7)	2 (3.9)	
KI67 ^a^			
Positive	89 (77.4)	51 (86.4)	0.154
Ignored	26 (22.6)	8 (13.6)	

† Missing information; * *p* -value –Chi-square or Fisher test.

## Discussion

The COVID-19 pandemic lead to a reduction of breast cancer diagnosis by 48.7% in the population studied, which correspond to the majority of women assisted by the public health system of Campinas. Women diagnosed were more often symptomatic and had a family history of breast cancer. Most cases were diagnosed in stages I or II, which did not change due to the pandemic. However, the frequency of luminal A-like tumors was lower.

In the pandemic period, diagnosis was more frequent in symptomatic women (79.7% versus 50.4%, *p* < 0.001) and those with palpable masses (79.7% versus 56.5%, *p* = 0.003). Most cases were diagnosed through diagnostic mammograms (81.4% versus 47.0%, *p* < 0.001). This was probably due to the interruption of screening activities, with priority access to cases of clinical suspicion. It is interesting to note that patients with a family history of breast cancer were also prioritized in the diagnosis (29.8% versus 9.6%, *p* < 0.001), indicating greater awareness in seeking out the health system.

The 48.7% reduction in breast cancer diagnosis was mainly at the expense of reducing screening, maintaining access to symptomatic patients. The number of cases diagnosed in symptomatic patients dropped from 58 to 47, a 23% reduction, and cases diagnosed through diagnostic mammograms dropped from 54 to 48, a 13% reduction. In fact, the proportion of in situ tumors, which represents the cases diagnosed through mammography, decreased from 21.7 to 13.6% in the period ( *p* = 0.193).

The literature already points out this profile of tumors diagnosed in symptomatic women during the pandemic. In the Netherlands, a significant reduction in diagnoses of in situ tumors and stage-I cases were observed in the pandemic period. 19 They also observed a more significant reduction in women aged 50 to 74 years, the target group for screening. 19 In our analysis, this influence of age on the decline was not observed, probably because women under 50 are also included in the opportunistic screening.

For symptomatic women, the mean days from symptom onset to mammography was 233.6 days (SD 458.3) in 2019 and 152.1 days (151.5) in 2020 ( *p* = 0.871). Although the difference was not significant, a possible time reduction may reflect easier access to symptomatic patients since the practice of regular screening was reduced. In Canada, a referenced care unit for breast cancer diagnosis changed its structure to rapid diagnostic unit during the pandemic and observed a significant reduction in the time between the mammogram and the final diagnosis. 20 The reduction of time to access the service and tests for diagnosis is essential for better efficiency of early diagnosis.

Among the invasive tumors, the stage profile was not different between the periods. The proportion of early breast cancers was slightly higher in the COVID period but not significant (76.7% in 2019 and 82.4% in 2020, *p* = 0.428). The subanalysis of the 110 cases diagnosed in stages I and II revealed that the pandemic seems to have selected the same pattern of cases for diagnosis, regardless of stage: cases were observed in symptomatic women and in those with familial history of breast cancer. An analysis from Turkey with 148 patients demonstrated increased tumor size and axillary involvement in the COVID period. 21 A more extended period of study is needed to assess any tendency in our sample.

In the COVID period, the frequency of luminal A-like tumors was lower (29.2% in 2019 and 11.8% in 2020, *p* = 0.018), as was the frequency of tumors with positive estrogen receptors (82.2% in 2019 and 66.0% in 2020, *p* = 0.030). The frequency of triple-negative tumors was twice as high in the COVID period, although not significantly (10.1% in 2019 and 21.6% in 2020, *p* = 0.062), probably due to the small number of cases analyzed.

The molecular subtypes of breast cancer are important because they show distinct pathogenic pathways and allow for individualized therapeutic approaches. Prognoses vary significantly depending on molecular types and access to treatment. About 70% of tumors are of the luminal type, with positive hormone receptors, when an estrogen receptor activates the tumor growth pathway. They are classified as A or B according to the absence (A) or presence (B) of the human epidermal growth factor 2 (HER2) protein overexpression and high expression of the K _i_ -67 protein. Tumors can also be hormone receptor-negative and HER2 positive, the pure HER2 types. They are triple-negative when they do not express any of these markers and have an unknown pathogenic pathway. 22 23

The reduction observed in luminal A-like tumors and the increase, albeit not significant, in triple-negative tumors indicate that the impact of the pandemic was more observed in the group of more indolent growth ones. This study showed that tumors with a more aggressive profile were the ones more frequently diagnosed. It supports the evidence that indolent tumors have greater sensitivity to screening. 24 25

The strength of this study was the availability of data in a high-quality information database, an uncommon event in low- and middle-income regions. As the service audited is the largest reference service in a populous city in Brazil, it reflects the population living in similar regions. The main limitation is the reduced observation time, which makes more robust analyses difficult due to the number of cases. It was also not possible to assess the evolution of the cases.

## Conclusion

During the COVID-19 pandemic, a reduction in breast cancer diagnoses was observed. Cases detected were suggestive of a worse prognosis: symptomatic women with palpable masses and more aggressive subtypes. Indolent tumors were those more affected by the interruption in screening.
